# The Effect of Cannabidiol in Conjunction with Radiation Therapy on Canine Glioma Cell Line Transplanted in Immunodeficient Mice

**DOI:** 10.3390/vetsci12080735

**Published:** 2025-08-05

**Authors:** Masayasu Ukai, Jade Kurihara, Markos Antonakakis, Krista Banks, Steve Dow, Daniel L. Gustafson, Mary-Keara Boss, Amber Prebble, Stephanie McGrath

**Affiliations:** 1 Department of Clinical Sciences, College of Veterinary Medicine and Biomedical Sciences, Colorado State University, Fort Collins, CO 80523, USA; masa.ukai@colostate.edu (M.U.); markos.antonakakis@colostate.edu (M.A.); steven.dow@colostate.edu (S.D.); daniel.gustafson@colostate.edu (D.L.G.); keara.boss@colostate.edu (M.-K.B.); 2 Brain Research Center, Colorado State University, Fort Collins, CO 80523, USA; 3 Flint Animal Cancer Center, College of Veterinary Medicine and Biomedical Sciences, Colorado State University, Fort Collins, CO 80523, USA; jade.kurihara@colostate.edu (J.K.); krista.banks@colostate.edu (K.B.); amber.prebble@colostate.edu (A.P.); 4 University of Colorado Cancer Center, Aurora, CO 80045, USA

**Keywords:** J3TBG, xenogeneic transplantation, CD-1 nude mice, CBD

## Abstract

Glioma is a type of neoplasia that spontaneously arises from the glial cells of the brain in humans and dogs, and its prognosis is grave. Current treatment options for glioma include surgery, radiation therapy, chemotherapy, or symptomatic treatment. Evidence has shown that cannabidiol (CBD) may have anticancer, anti-angiogenic, and anti-inflammatory properties in both in vitro and in vivo studies. In this in vivo murine experiment, the canine glioma cell line J3TBG was injected into the frontoparietal cortex of immunodeficient mice using xenogeneic tissue transplantation. A total of 20 mice were randomly assigned to one of four treatment groups—Control group (C), CBD group (CBD), Radiation Therapy group (RT), and CBD plus Radiation Therapy group (CBD + RT). After transplantation of J3TBG, a single fraction of 5.5 Gy RT was administered to the RT and CBD + RT groups, and CBD was administered daily to the CBD and CBD + RT groups. Necropsies were performed to collect blood and brain tissue. Although there was not a statistically significant difference, the survival time among mice were longer in the CBD + RT group than the RT group. These results indicate that CBD may be used as an adjunctive therapy to enhance RT treatment. Larger cohort studies are required to substantiate the hypothesis.

## Simple Summary

Glioma is a type of brain tumor that occurs in humans and dogs. Its prognosis is grave, with current treatment regimens such as surgery, radiation therapy, chemotherapy, or symptomatic treatment. Cannabidiol (CBD) has drawn attention since it may have anticancer properties against different tumors including glioma. In this in vivo murine experiment, the canine glioma cell line J3TBG was injected into the cerebral cortex of immunodeficient mice. Twenty mice were randomly assigned to one of four treatment groups—Control group (C), CBD group (CBD), Radiation Therapy group (RT), and CBD plus Radiation Therapy group (CBD + RT). A single fraction of RT was given to the RT and CBD + RT groups, and CBD was administered daily to the CBD and CBD + RT groups. The survival time among mice were longer in the CBD + RT group than the RT group, although it was not statistically significant. These results indicate that CBD may be used as an adjunctive therapy to enhance the effect of radiation treatment.

## 1. Introduction

Glioma is a type of neoplasia that spontaneously arises from the glial cells of the brain or spinal cord in humans and dogs. Glioma may occur at any place in the central nervous system due to the distribution of glial cells—astrocytes and oligodendrocytes. Glioma can be classified into high and low grades based on the morphic population of neoplastic cells, mitotic rate, necrosis and microvascular proliferation [1,2,3]. Prognosis of glioma is grave; in humans with high-grade gliomas, the median survival time is 14–16 months, while 2–4 months in dogs [4,5].

Current treatment for glioma includes debulking surgery, radiation therapy (stereotactic radiation therapy or conventionally fractionated radiation therapy), chemotherapy, combination therapy, or symptomatic treatment with/without palliative radiation therapy [6]. Unfortunately, outcomes have not improved in tandem with the medical advances in treatment protocols for gliomas [7].

Cannabinoids are a family of naturally occurring compounds that are extracted from the cannabis plant, with cannabidiol (CBD) being the most abundant [8]. Evidence has shown that CBD may have anticancer, anti-angiogenic, anti-inflammatory, and anti-seizure properties in both in vitro and in vivo studies [9,10,11,12,13,14,15,16], which have drawn attention. Studies have shown potential cytotoxic, anti-proliferative, and anti-migratory effects of CBD in various type of neoplasms in vitro [17,18,19].

A recent study revealed that CBD demonstrated a range of cytotoxic effects on both human and canine glioma cells with, canine glioma cells showing higher sensitivity [20]. Moreover, the mechanism of action behind this may relate to the dysregulation of calcium homeostasis and mitochondrial activity [20].

Canine gliomas share many characteristics with humans, such as spontaneous occurrence, magnetic resonance imaging (MRI) findings, histopathological features, and immunological responses [1,21,22], which indicate that canine glioma can serve as an animal model for human glioma research. The objective of this study was to investigate the anticancer effect of CBD on canine glioma cells transplanted in mice brain with and without concurrent radiation treatment. We hypothesized that administration of CBD would enhance the effect of radiation therapy on canine glioma cells.

## 2. Material and Methods

In this in vivo murine experiment, 14-day-old female CD-1 nude mice were purchased from Charles River Laboratories (Wilmington, MA, USA). The canine glioma cell line J3TBG transfected with luciferase was injected into the frontoparietal cortex of immunodeficient mice using xenogeneic tissue transplantation. A total of 20 mice were divided into four treatment groups. Five mice were randomly assigned to each treatment group using Microsoft Excel (Microsoft: Redmond, WA, USA)—Control group (C), CBD group (CBD), Radiation Therapy group (RT), and CBD plus RT group (CBD + RT). The Ethics Committee for Animal Use of IACUC approved the research under the protocol #2937. The data that support the findings of this study are available from the corresponding author upon reasonable request.

### 2.1. In Vivo Xenogeneic Orthotopic Glioma Transplantation

Canine glioma cell line J3TBG expressing luciferase was suspended in phosphate-buffered saline (PBS) at a concentration of 1.0 × 10^6^ cells/mL.

Glioma cell transplantation was performed using a previously described method [23]. Mice were anesthetized with isoflurane (Isospire: Dechra, Northwich, UK); the injection site was clipped, and the skin was prepared with alternating betadine and 70% EtOH wipes. Preoperatively, the mice were given a single dose of buprenorphine, released slowly (Buprenorphine Hydrochloride Injection: PAR pharmaceutical, Woodcliff Lake, NJ, USA) at a dose of 0.6–0.8 mg/kg subcutaneously, for pain management. A midline sagittal skin incision was made using a blade. Mice were mounted onto a stereotactic immobilization system (Just for Mouse Stereotaxic Instrument: Stoelting Co., Wood Dale, IL, USA). Sterile cotton swabs were used to remove the membranes to reveal the bregma suture. A craniotomy was performed near the bregma suture, and a Hamilton syringe with the J3TBG tumor cells was lowered into the frontoparietal cortex via the craniotomy site. A solution containing 100,000 J3TBG cells eluted in a volume of 3 μL were injected in 1 μL boluses, waiting one minute between injections. Mice were removed from the device, and the skin was closed with interrupted sutures (surgical suture, 6–0 monofilament, non-absorbable) and surgical glue (Cyanoacrylate, Vetbond: 3M, Saint Paul, MN, USA). All mice recovered from anesthesia uneventfully.

### 2.2. Radiation Therapy (RT)

The ten mice in the RT and CBD + RT groups were anesthetized and irradiated with 5.5 Gy ionizing radiation five days after xenogeneic glioma cell implantation. The remaining ten mice in the C and CBD groups were anesthetized as controls, but the tumors were not irradiated. RT beams were administered with an X-RAD SmART+ irradiator (Precision X-ray, Inc., North Branford, CT, USA) and collimated to include only the region of the brain containing the gross neoplasm. Proper alignment of the treatment field was confirmed with two fluoroscopic images taken at 0° and 270° using the X-RAD SmART+ irradiator. After the RT procedure, all mice including C and CBD groups received dexamethasone SP (dexamethasone SP injection: Veton, Boise, ID, USA) at a dose of 0.0012 mg per mouse intra-peritoneally (IP) every 24 h during the study (until euthanasia).

### 2.3. CBD IP Injection

The ten mice in the CBD and CBD + RT groups were given CBD liquid IP, and the remaining ten mice in the C and RT groups were given the CBD vehicle IP at an equivalent volume. The vehicle comprised Cremophor EL: Ethanol: Saline in a 1:1:18 ratio. CBD liquid was the vehicle with CBD powder (CBD Isolate, Hemp Derived: Extract Labs, CO, USA) added to achieve a 30 mg/kg dose every 24 h.

### 2.4. IVIS Imaging for Monitoring Glioma Size in the Brain

An IVIS machine provides semi-quantitative images in which signal intensities correlate positively with the number of glioma cells that express luciferase. In order to visualize the neoplasm, the mice were injected with luciferin 0.2 mL per mouse in the dorsal cervical region subcutaneously and subsequently anesthetized with isofluorane twice per week. They were placed in IVIS Spectrum (PerkinElmer, Inc, Waltham, MA, USA) for imaging. The total time under anesthesia was approximately 10 min per mouse.

### 2.5. Monitoring for Short-Term and Long-Term Effects

The size of the neoplasms was expected to be correlated with behavioral changes, neurological signs, or seizures. Expected neurological signs included altered mentation, circling, head turn, incoordination of gait, and expected behavioral changes were decreased interaction with other mice and aggressive behavior. Neurological signs or behavioral changes that corresponded with increased morbidity resulted in immediate euthanasia of the mice. The method of euthanasia is described in the next section.

### 2.6. Harvesting of the Blood and Brain for CBD Concentration and Histopathology

When the mice developed severe neurological signs or subcutaneous neoplasia grew beyond 0.5 × 0.5 × 0.5 cm^3^ (0.125 cm^3^) or if the quality of life was affected such as impaired vision, they were euthanized via cervical dislocation, and necropsies were performed to collect blood and brain tissue. In the CBD and CBD + RT groups, the mice were given a final CBD injection, and within four hours, approximately 0.8–1.0 mL of blood was extracted via cardiac puncture under anesthesia with isoflurane. Subsequently, the mice were euthanized using cervical dislocation. Plasma was isolated and stored for CBD concentration [20]. One half of each brain and neoplasm was placed in an Eppendorf tube and frozen for tissue CBD concentration detection, and the other half was placed in formalin, paraffin-embedded, and then sliced at 5 μm thickness for histopathological examination. Slides containing glioma, normal cerebrum, brainstem, and cerebellum were stained with hematoxylin and eosin (H&E) and Ki-67 for immunohistochemistry. The slides were imaged using an Olympus slide scanner (VS200: Olympus, Shinjuku City, Japan). Positive cell count was performed on the slides stained with Ki-67 via Olivia (Olympus software: Olympus, Shinjuku City, Japan) and divided by the region of interest used, resulting in the total number of positive cells/mm^2^.

### 2.7. CBD Extraction Procedure for Plasm

Cannabidiol was stored at −20 °C as a 1 mg/mL solution in methanol, and D3 Cannabidiol (D3-CBD) was stored at −20 °C as a 100 μg/mL solution in methanol. Initial stock solutions, 1 mg/mL in methanol (Millipore Sigma, Burlington, MA, USA), were prepared in 1.5 mL microcentrifuge tubes and stored at −20 °C. The liquid–liquid extraction procedure for curve, quality control (QC), and submitted tear samples was performed as follows: A 200 ng/mL D3-CBD solution was prepared in acetonitrile as an internal standard for the precipitation reagent. Standard dilutions of cannabidiol (0.98, 1.95, 3.9, 7.8, 15.6, 31.25, 62.5, 125, 250, 500, 1000, and 2000 ng/mL) were prepared in acetonitrile as a 10× working stock. Then, 5 μL of each 10× standard was added to a fresh 1.5 mL microcentrifuge tube for curve. Next, 50 μL of blank mouse plasma was added to each standard tube. QC samples were prepared by adding 5 μL of the designated standard 10× concentrate, with n = 3 at each concentration. Investigative plasma samples were prepared by transferring 50 μL of sample to each prelabeled corresponding extraction tube, to which 5 μL of acetonitrile was added. All samples received 5 μL of acetonitrile containing 200 ng/mL d3-CBD. The curve standard, QC, and investigative samples received 1000 μL of ethyl acetate. Samples were vortexed for 10 min and then centrifuged at 14,000 rpm for 10 min. Then, 900 μL of the organic phase was transferred to fresh 1.5 mL microcentrifuge tubes. All samples were evaporated to dryness in rapidvap vertex (Labconco, MO, USA) for about 20 min. All samples were reconstituted in 50 μL of 75%/25% acetonitrile with 0.1% formic acid and milli-Q water with 0.1% formic acid. Samples were shaken for 10 min and then centrifuged at 14,000 rpm for 1 min. Next, 50 μL was transferred to vials with glass inserts for analysis using Liquid Chromatography–Mass Spectrometer (LCMS). LCMS was performed with an injection volume of 5 μL, a flow rate of 1000 μL/min, a run time of 7.0 min, and a column oven of 30 °C.

### 2.8. CBD Extraction Procedure for Brain Samples

Brain from an untreated mouse (1 part) was homogenized in 9 parts milli-Q water to use as the matrix for the curve and QC samples. Submitted brain samples were weighed, and milli-Q water was added at a 9-fold volume, and samples were homogenized. A standard 10× calibration curve was created using 2-fold dilutions of concentrated analyte, starting at 20,000 ng/mL. Subsequent dilutions were 10,000, 5000, 2500, 1250, 625, 312.5, 156, 78, 39, 19.5, and 9.8 ng/mL as 10× concentration additives in acetonitrile. D3-CBD was diluted in acetonitrile to 2000 ng/mL for use as a 10× concentration internal standard. For the standard curve and QC samples, 100 microliters of blank mouse brain homogenate was transferred to each prelabeled 1.5 mL polypropylene tube for extraction. For each corresponding standard, 10 μL of 10× standard was added. For submitted brain samples, 100 μL of experimental brain homogenate was transferred to correspondingly labeled tubes along with 10 μL of acetonitrile. Each extraction tube received 10 μL of 10× internal standard (IS) for a final IS concentration of 200 ng/mL. Each sample received 1000 μL of ethyl acetate. Samples were quickly vortexed to mix, and then transferred to a vortex shaker for a minimum of 10 min at room temperature. Samples were spun at 14,000 rpm for 10 min, and 900 μL of organic phase was transferred to fresh tubes to be dried down using a speedvac at high heat for 60 min. Samples were reconstituted in 50 μL of 75%/25% acetonitrile with 0.1% formic acid and milli-Q water with 0.1% formic acid, vortexed well, and then centrifuged for 1 min at 14,000 rpm. Reconstituted samples were transferred to prelabeled vials with polypropylene inserts for analysis by Liquid Chromatography–Mass Spectrometer (LCMS). LCMS was performed with an injection volume of 5 μL, a flow rate of 1000 μL/min, a run time of 7.0 min, and a column oven of 30 °C.

### 2.9. Statistical Analysis

Descriptive and analytical statistics were performed. Data used for analytical statistical analysis included survival time, the number of positive cells on Ki-67-stained samples, and CBD plasma concentration. All numerical variables obtained were evaluated for normality and variance and based on the result, calculated using either the two-sample *t*-test, the paired *t*-test, Welch’s test, the Mann–Whitney test, One-way Anova, or the Kruskal–Wallis test with RStudio version 1.1.463 (RStudio, PBC, Boston, MA, USA). Furthermore, post hoc analyses with either the pairwise *t*-test or the Dunn test were performed to identify where a significant difference occurred, if the One-way Anova or the Kruskal–Wallis test demonstrated significant difference among the groups. Statistical significance was defined as *p* < 0.05.

## 3. Results

**Figure 1:**
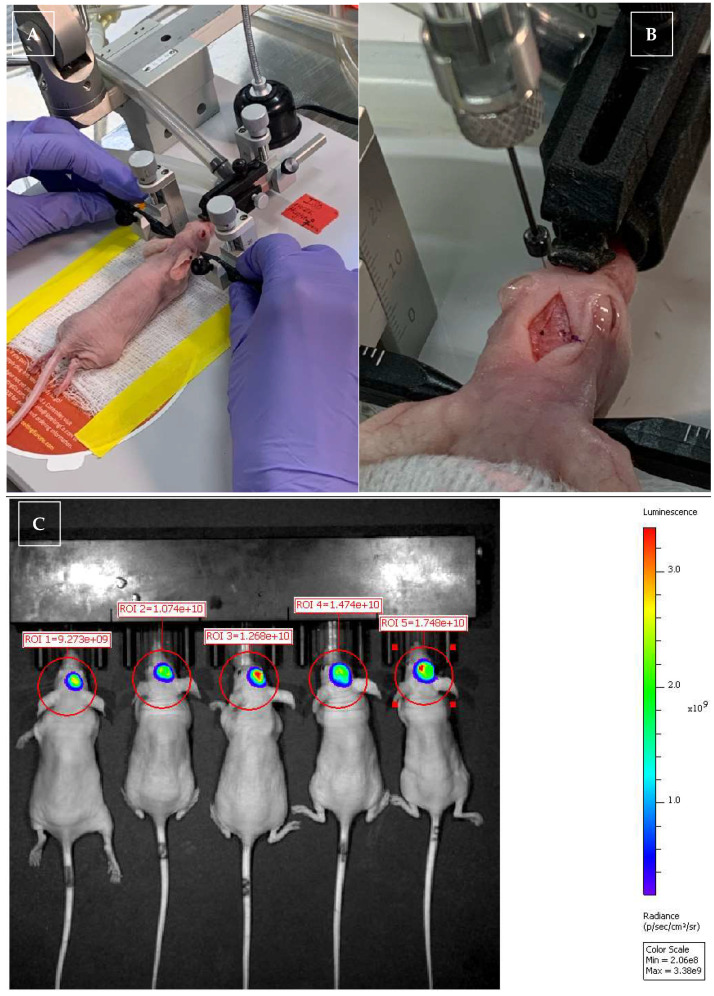

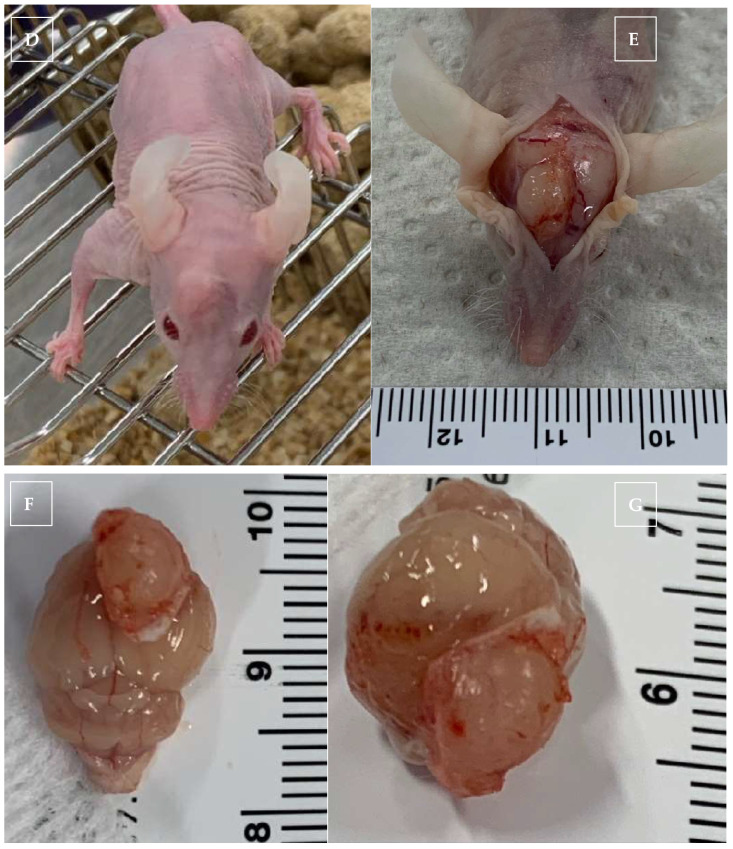
Pictures of glioma implantation and IVIS imaging for monitoring glioma size, and gross pictures of the J3TBG glioma that was implanted and growing in the mice brain and subcutaneous area. Picture (**A**,**B**): During in vivo xenogeneic orthotopic glioma transplantation. Mice were mounted onto a stereotactic immobilization system (**A**), and a craniotomy was performed—the marker on the midline is bregma (**B**). IVIS imaging was collected twice per week to monitor glioma during the study period (**C**). Picture of a mouse after implanted tumor growth and before euthanasia due to tumor size (**D**). Pictures of necropsy: skin incision (**E**); brain extracted with a dorsal-to-ventral view, with the cranial aspect of the brain located at the top of the picture (**F**); and brain extracted with a dorsal-to-ventral view, with the cranial aspect of the brain located at the bottom of the picture (**G**). A grown tumor is visible in the frontoparietal lobe (**F**,**G**).

Descriptive statistical results from all groups are shown in Table 1, and gross pictures of mice and harvested brain are shown in Figure 1. There were no complications directly related to daily CBD or vehicle IP injections, RT, or anesthesia.

**Figure 2:**
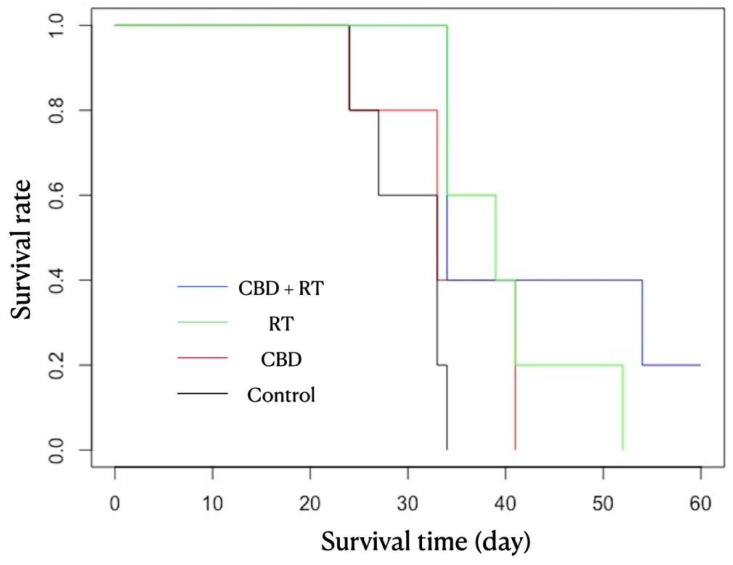
The Kaplan–Meier curves for different groups. Black—Control group; red—Cannabidiol (CBD) group; green—Radiation Therapy (RT) group; blue—CBD + RT group. Significant differences in survival time were found between the C and RT groups and between the C and CBD + RT groups (log-rank test; *p* = 0.009 and *p* = 0.01, respectively).

All mice were euthanized by Day 55 after the transplantation due to neurological signs or subcutaneous neoplastic growth. In Group C, all mice were euthanized by Day 34 due to subcutaneous neoplastic growth, including one at Day 24, one at Day 27, two at Day 33, and one at Day 34. In the CBD group, all mice were euthanized by Day 41 due to subcutaneous neoplastic growth in all mice, except for one that demonstrated neurological signs (circling and body twitch), including one at Day 24, two at Day 33, and two at Day 44. In the RT group, all mice were euthanized by Day 52 due to subcutaneous neoplastic growth, including two at Day 34, one at Day 39, one at Day 41, and one at Day 52. In CBD + RT group, four mice were euthanized by Day 54 due to subcutaneous neoplastic growth, including three at Day 34 and one at Day 54, but one other mouse was euthanized at Day 60 to cease this study since the mouse never demonstrated any neurological signs nor exhibited subcutaneous neoplastic growth. As the statistical analysis in Figure 2 shows, there were significant differences in survival time between the C and RT groups and between the C and CBD + RT groups (log-rank test; *p* = 0.009 and *p* = 0.01, respectively).

For CBD concentration shown in Table 2, plasma, neoplasm, and non-diseased brain were examined separately. Plasma CBD concentration had a mean of 181.6 ng/mL and a median of 181 ng/mL (range 158–208) in the CBD group, and a mean of 145.2 ng/mL and a median of 163 ng/mL (range 101–167) in the CBD + RT group. CBD concentration in the neoplasm had a mean of 355.6 ng/mL and a median of 376 ng/mL (range 182–496) in the CBD group, and a mean of 258.68 ng/mL and a median of 320 ng/mL (range 93.4–365) in the CBD + RT group. CBD concentration in non-diseased brain tissue had a mean of 403.2 ng/mL and a median of 456 ng/mL (range 233–579) in the CBD group and a mean of 269.2 ng/mL and a median of 253 ng/mL (range 176–379) in the CBD + RT group. There was no detectable CBD in plasma, neoplasm, and non-diseased brain tissue in the C and RT groups.

Statistical analysis in both the CBD and CBD + RT groups showed significant differences in CBD levels between plasma and neoplasia (*p* = 0.003, 95% CI = 60.4–227, paired *t*-test) and between plasma and intact brain (*p* = 0.0007, 95% CI = 94.6–250.9, paired *t*-test), but no significant difference between neoplasia and non-diseased brain tissue (*p* = 0.439, 95% CI = −110.3–52.2, paired *t*-test).

**Figure 3:**
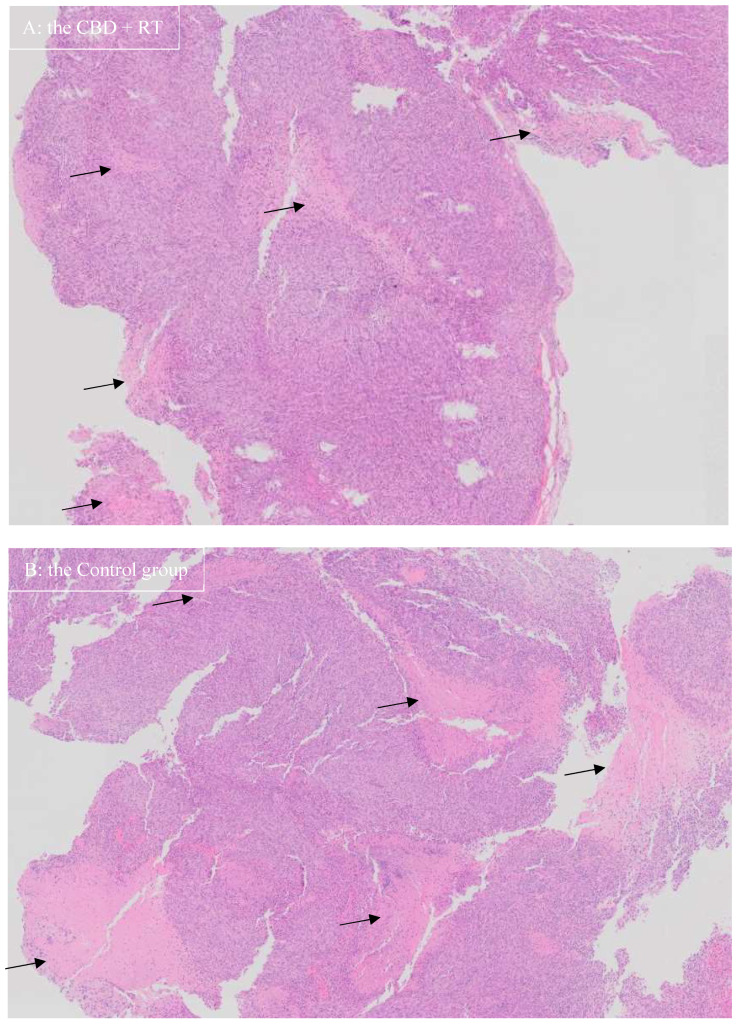
Hematoxylin and eosin stain of J3TBG glioma in the mice brain that shows highly invasive nature: the picture on the top (**A**) from a mouse in the CBD + RT group, the picture on the bottom (**B**) from a mouse in the Control group (magnification ×2). Necrotic areas (black arrows) are more pronounced in the Control group than in the CBD + RT group.

**Figure 4:**
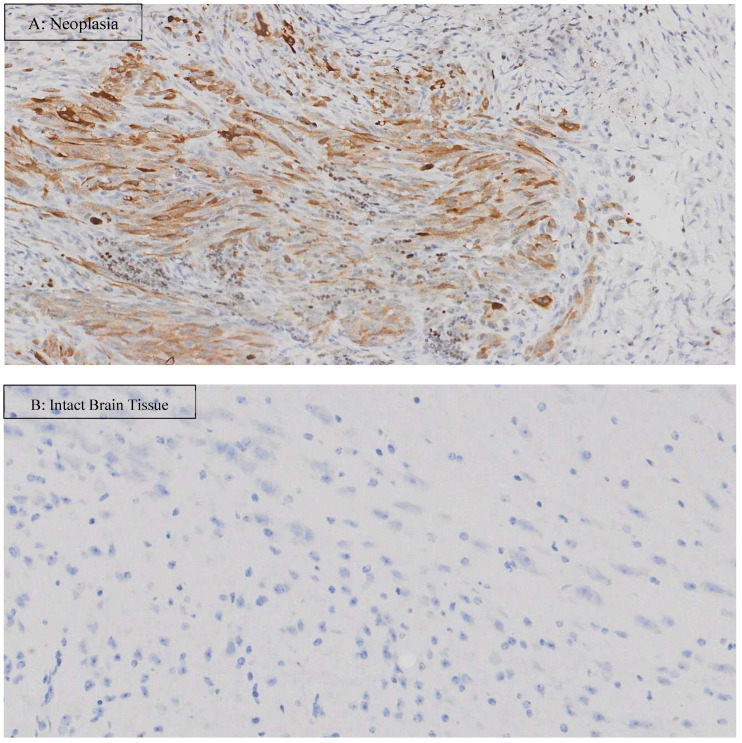
Ki-67 staining of J3TBG glioma in mice brain. The picture on the top (**A**) shows neoplasia, and the picture on the bottom (**B**) shows intact brain tissue in the CBD group (magnification ×20). Ki-67-positive cells that are stained brown are illustrated in the top picture (the neoplasia) but not in the bottom picture (the intact brain tissue).

For histopathological examination, representative H&E microscopic images are shown in Figure 3. The appearance of the neoplasia looked similar among groups; however, necrotic areas appeared more pronounced in Group C than the other groups where CBD + RT group showed the mildest necrosis. The percentage of Ki-67-positive cells over total nuclei cells (%) and the number of positive cells (cells/μm^2^) are shown in Table 3. Representative images of Ki-67 staining are shown in Figure 4. In Group C, positive nuclei mean (median) was 0.235 (0.177)% and 15.364 (10.866)/μm^2^ in the neoplasm and 0.051 (0.051)% and 1.427 (1.420)/μm^2^ in non-diseased brain tissue. In the CBD group, positive nuclei mean (median) was 1.124 (0.774) % and 62.336 (47.833)/μm^2^ in the neoplasm and 0.620 (0.342)% and 16.086 (9.070)/μm^2^ in non-diseased brain tissue. In the RT group, positive nuclei mean (median) was 1.590 (1.282)% and 95.587 (64.529)/μm^2^ in the neoplasm and 0.485 (0.370)% and 11.927 (8.374)/μm^2^ in non-diseased brain tissue. In the CBD + RT group, positive nuclei mean (median) was 0.511 (0.531)% and 27.999 (27.658)/μm^2^ in the neoplasm and 0.218 (0.128)% and 5.009 (3.071)/μm^2^ in non-diseased brain tissue.

Statistical analysis did not reveal any significant differences among the four groups with respect to the percentage of Ki-67-positive nuclei (%) and the number of positive cells (cells/μm^2^) in the neoplasm and non-diseased brain tissue.

## 4. Discussion

Humans and dogs both develop naturally occurring gliomas, with similar clinical signs, biological and histopathological features, diagnostic imaging findings and poor prognosis [21,24,25,26]. In terms of treatment, adjunctive treatments, such as temozolomide, paclitaxel, and immune-modulatory nanoparticles, have been researched *in vitro* and *in vivo*, showing potential benefits [4,27,28,29,30]. Research into alternative treatments using canine glioma cell lines may not only help improve dogs but also contribute to human medicine, as canine glioma is considered a relevant large-animal model for human glioma [21,24,25,26].

Canine glioma cell lines have been researched to explore better treatment options [27,28,31,32,33]. The canine glioma cell lines, J3TBG, G06-A, and SDT-3G, have been established as canine high-grade glioma and have been used both *in vitro* and *in vivo* studies, including xenogeneic transplantation [27,28,31,32,33]. J3TBG cells are known to grow both orthotopically and heterotopically in a xenogeneic immunodeficient host such as CD-1 nude mice, and can infiltrate surrounding tissue, but less invasively than in allogeneic dog hosts [32]. This line has been successfully utilized for postnatal implantation in the brain of adult animals to generate an anaplastic astrocytoma model similar to that in human patients, as well as for induction of immune tolerance in fetal beagle dogs [34]. A recent study investigating the sensitizing effect of CCNU alone, temozolamide alone, and their combination with irradiation (4 Gy) in J3TBG [27] cells revealed that combining CCNU and temozolamide with radiation significantly reduced J3TBG glioma cell survival, even after long-term drug exposure that leads to the generation of either CCNU- or temozolamide-resistant cells. To the authors’ knowledge, a study on CBD’s efficacy on these glioma cell lines has not yet been conducted; thus, this is the first paper to investigate it using J3TBG cell lines.

We performed this study to investigate the anticancer effect of CBD on canine glioma cells transplanted in the immunodeficient mouse brain, with and without concurrent radiation treatment. Although there was no statistically significant difference, the survival time among mice were longer in the CBD + RT group than the RT group and shorter in the CBD group than the RT group, as shown in Figure 2. These results indicate that although CBD may not be as effective as RT alone, it may help as an adjunctive therapy to enhance RT treatment. The increased survival time in the CBD + RT group compared to the RT group might be attributable to the anticancer effect of CBD or CBD’s role as a radiosensitizer in glioma. The potential reason for no statistically significant difference could be underpowering due to a low number of sample sizes. Based on the post hoc analysis with the Cox proportional hazard model, 71 mice per group would be required to establish statistical significance between the RT group and the CBD + RT group, and 42 mice per group would be required to establish significance between the CBD group and the RT group. In order to establish this trend of CBD effect, larger cohort studies are needed.

CBD has been researched *in vitro* and *in vivo*, demonstrating its potential anticancer effects [35,36]. CBD is considered to induce production of reactive oxygen species (ROS), glutathione (GSH) depletion, and caspase-9, -8, and -3 activation, and induction of apoptosis. Indeed, cannabinoids have shown significant reduction in tumor growth in subcutaneous and orthotopic glioma transplantation using animal models [37]. In most studies, agonistic stimulation via CB receptors is responsible for the anticancer properties of cannabinoids, which suggests that CB1 agonists may be useful in glioma therapy.

In the present study, CBD concentrations in plasma, non-diseased brain tissue, and glioma tissue were measured. The median plasma CBD concentration was 181 ng/mL in the CBD group and 163 ng/mL in the CBD + RT group. The median glioma CBD concentration was 376 ng/mL in the CBD group and 320 ng/mL in the CBD + RT group. For non-diseased brain tissue, the median was 456 ng/mL in the CBD group and 253 ng/mL in the CBD + RT group. In both the CBD and CBD + RT groups, there was a significant difference in the CBD levels in plasma vs. neoplasm and plasma vs. non-diseased brain, but not between neoplasm vs. non-diseased brain tissue. We had expected that the neoplasm would have higher CBD concentrations than non-diseased brain tissue due to increased vascularization of neoplastic tissues. In one CBD PK study in mice, cremophor-based CBD, which is similar to the CBD used in the present study, was given IP at a dose of 120 mg/kg, and CBD concentration was measured in the plasma and brain. The Cmax plasma and brain tissue CBD concentration was 14.3 μg/mL in 2 h (Tmax) and 6.9 μg/mL in 1 h (Tmax), with similar half-life of around 4.5 h [38]. In the present study, 30 mg/kg CBD was injected IP, and blood, non-diseased brain ,and glioma were harvested within 4 h of final injection for each mouse. With simple calculation of 30 mg/kg CBD IP assuming presence of a linear relationship, the concentration should be 357 ng/mL in plasma and 170 ng/mL in brain based on this study. Our study revealed more than 250 ng/mL of CBD concentration in the non-diseased brain and glioma, which is a much higher level than the previous PK study. On the other hand, the concentration in plasma in the present study was significantly lower than in the previous study. This difference in CBD levels in the brain and plasma between the previous PK study and our study could be explained by the timing of extraction.

In the present study, the percentage of Ki-67-positive cells over total nuclei cells (%) and the number of positive cells (cells/μm^2^) were calculated. A meta-analysis of human glioma in 2015 revealed that Ki-67 expression might be a predictive factor for poor prognosis in glioma patients [39]. Although the glioma cell line was the same in the present study, it was assumed that Ki-67-positive cells in Group C would be greater than in the other groups; however, the percentage of Ki-67-positive cells over total nuclei cells (%) and the number of positive cells (cells/μm^2^) in Group C were fewer than in the other groups. The reason for this is not known, but since the J3TBG glioma cell line is known to behave aggressively, there might be a contribution of more necrotic tumor cells generated in Group C than in the treatment groups (CBD, RT, CBD + RT groups), resulting in fewer percentage of Ki-67-positive cells and fewer number of positive cells (cells/μm^2^), as shown in Figure 3. Among treatment groups, the RT + CBD group demonstrated the lowest values for both the percentage and number of Ki-67-positive cells, which may represent the usefulness of Ki-67 for treatment evaluation. Further studies are required to assess utility of Ki-67 for the J3TBG glioma cell line.

Glioma is a major health challenge due to the current lack of effective treatments, most likely because the blood–brain barrier interferes with drug delivery to the central nervous system. Consequently, high doses of chemotherapy are typically required to attain therapeutic levels in the brain, resulting in severe toxicity to other organs. Thus, surgery and/or RT are often elected for glioma treatment. Thus far, monotherapy and combination therapy have been deemed ineffective in establishing a reasonable prognosis in humans and dogs. CBD might help develop effective strategies for drug delivery to the brain and overcome biodistribution and PK limitations of conventional chemotherapy agents since CBD has the capability of high BBB transcytosis [36].

In conclusion, CBD as an adjunctive therapy appeared to increase survival time in this study compared to RT alone. To further demonstrate CBD’s efficacy on gliomas in dogs and humans, larger cohort studies are required using different glioma cell lines such as G06-A and SDT-3G, as well as clinical trials in dogs with naturally occurring disease. Canine glioma is considered to be a relevant animal model of human disease. Urgent research on this field is critical to establish better outcomes of glioma treatment in veterinary and human medicine.

## Figures and Tables

**Table 1:** Descriptive statistical analysis of the results from all 20 mice.

Group	Mouse	CBD/Vehicle IP Injection Daily	RT	Neurological Signs	Survival Time (day)	Mean (Median) Survival Time
Control	1	Vehicle	n/a	-	33	30.2 (33)
	2			-	33	
	3			-	34	
	4			-	24	
	5			-	27	
CBD	1	CBD 30 mg/kg q24 h	n/a	-	24	34.4 (33)
	2			+	33	
	3			-	41	
	4			+	41	
	5			-	33	
RT	1	Vehicle	5.5 Gy once	-	34	40 (39)
	2			-	52	
	3			-	41	
	4			-	39	
	5			-	34	
CBD + RT	1	CBD 30 mg/kg q24 h	5.5 Gy once	-	34	43.2 (34)
	2			-	34	
	3			-	54	
	4			-	60	
	5			-	34	

**Table 2:** Cannabidiol (CBD) concentration level in plasma, intact brain, and neoplasia among 4 groups. RT: radiation therapy.

Group	Mouse	CBD Level in Plasma (ng/mL)	Mean (Median) (ng/mL)	CBD Level in Neoplasia (ng/mL)	Mean (Median) (ng/mL)	CBD Level in Intact Brain (ng/mL)	Mean (Median) (ng/mL)
Control	1	0	0 (0)	0	0 (0)	0	0 (0)
	2	0		0		0	
	3	0		0		0	
	4	0		0		0	
	5	0		0		0	
CBD	1	181	181.6 (181)	496	355.6 (376)	579	403.2 (456)
	2	208		376		471	
	3	158		182		277	
	4	166		257		233	
	5	195		467		456	
RT	1	0	0 (0)	0	0 (0)	0	0 (0)
	2	0		0		0	
	3	0		0		0	
	4	0		0		0	
	5	0		0		0	
CBD + RT	1	101	145.2 (163)	181	258.68 (320)	176	269.2 (253)
	2	167		320		250	
	3	163		93.4		379	
	4	164		334		253	
	5	131		365		288	

**Table 3:** The percentage of Ki-67-positive cells over total nuclei cells (%) and the number of positive cells (cells/μm^2^). SD: standard deviation.

Group	Mouse	Neoplasia				Intact Brain Tissue			
		Positive Nuclei%	Mean (Median)	Positive Cell number/μm^2^	Mean (Median)	Positive Nuclei	Mean (Median)	Positive Cell Number/μm^2^	Mean (Median)
Control	1	0.201	0.235 (0.177) SD 0.291	14.580	15.364 (10.866) SD 18.893	0.037	0.051 (0.051) SD 0.010	1.060	1.427 (1.420) SD 0.256
	2	0.736		47.637		0.049		1.411	
	3	0.177		10.886		0.051		1.420	
	4	0.016		1.228		0.065		1.781	
	5	0.046		2.492		0.051		1.460	
CBD	1	3.049	1.124 (0.774) SD 1.097	158.374	62.336 (47.833) SD 56.198	0.245	0.620 (0.342) SD 0.733	5.733	16.086 (9.070) SD 18.384
	2	0.774		60.226		0.447		12.841	
	3	0.811		47.833		1.916		48.444	
	4	0.707		27.120		0.150		4.340	
	5	0.280		18.125		0.342		9.070	
RT	1	3.589	1.590 (1.282) SD 1.196	223.949	95.587 (64.529) SD 77.196	0.108	0.485 (0.370) SD 0.446	3.269	11.927 (8.374) SD 10.516
	2	0.586		35.399		1.254		30.236	
	3	1.282		64.529		0.272		8.180	
	4	0.808		44.746		0.422		9.576	
	5	1.686		109.311		0.370		8.374	
CBD + RT	1	0.632	0.511 (0.531) SD 0.217	47.674	27.999 (27.658) SD 17.218	0.050	0.218 (0.128) SD 0.220	1.344	5.009 (3.071) SD 4.498
	2	0.531		27.658		0.595		12.482	
	3	0.408		18.630		0.098		2.322	
	4	0.207		4.835		0.221		5.828	
	5	0.775		41.200		0.128		3.071	

## Data Availability

The data used and analyzed in the current study are available from the corresponding author upon reasonable request.
